# Supplementary material for: Long-Term Outcomes of Robotic-Assisted, Video-Assisted and Open Surgery in Non-Small Cell Lung Cancer: A Matched Analysis
Source: J Clin Med. 2022 Jun 11;11(12):3363. doi: 10.3390/jcm11123363 (PMC9225497; doi:10.3390/jcm11123363)
Supplement: Supplementary file 1 [file jcm-11-03363-s001.zip › jcm-1732768-supplementary.pdf]

Supplementary Table S1. Patients' characteristics (unmatched series, excluding conversions)

		Total	Open	VATS	RATS	Open vs VATS†	Open vs RATS†	VATS vs RATS†
		561	258	49	254			
**Age, years**, median [range]		67 [38–84]	68 [45–83]	67 [38–81]	67 [38–84]			
**Age group**	<60	119	51	13	55			
	60–64	98	44	5	49			
	65–69	133	62	13	58			
	70–74	120	54	12	54			
	75+	91	47	6	38	0.51	0.83	0.56
**Sex**	Male	311	146	23	142			
	Female	250	112	26	112	0.21	0.88	0.25
**ASA score**	1	26	14	2	10			
	2	436	185	42	211			
	3	84	54	5	25	0.16	**0.002**	1.00
	Missing	13	5	0	8			
**Clinical Stage**	Stage Ia1	94	20	8	66			
	Stage Ia2	231	100	19	112			
	Stage Ia3	140	84	14	42			
	Stage Ib	96	54	8	34	0.27	**<0.0001**	0.15

† According to the univariate conditional logistic regression score test. Bold text indicates a statistically significant difference with a p-value less than 0.05.
